# Vaccination against Bacterial Mastitis in Sheep

**DOI:** 10.3390/vaccines10122088

**Published:** 2022-12-07

**Authors:** Natalia G. C. Vasileiou, Daphne T. Lianou, Charalambia K. Michael, George C. Fthenakis, Vasia S. Mavrogianni

**Affiliations:** 1 Faculty of Animal Science, University of Thessaly, 41110 Larissa, Greece; 2 Veterinary Faculty, University of Thessaly, 43100 Karditsa, Greece

**Keywords:** health management, mastitis, milk production, sheep, *Staphylococcus*, udder, vaccination

## Abstract

The objective of this review is to discuss the application of vaccination for the prevention of bacterial mastitis in ewes, performed within the frame of health management schemes in sheep flocks. Mastitis is a multi-faceted infection, caused most often by staphylococci; hence, special emphasis is given to staphylococcal mastitis, also given that most relevant studies refer to vaccinations against that infection. Studies regarding various vaccines have been performed; most studies refer to vaccination by using a vaccine making use of cell-free surface polysaccharides in various vehicles, bacterial unbound cells or bacterial cells embedded in their biofilm matrix. Vaccination against mastitis should be better performed during the final stage of pregnancy to allow protection of ewes from lambing and should be considered as one of many control measures for the prevention of the disease. The expected benefits of mastitis vaccination in sheep flocks include the following: (a) reduced incidence risk of clinical and subclinical mastitis, (b) reduced somatic cell counts, optimum chemical composition, absence of staphylococci in milk, (c) increased milk production, (d) reduced dissemination of mastitis-causing pathogens and (e) reduction of antibiotic use in flocks.

## 1. Introduction

Sheep farmers strive to provide high-quality products from their animals, at the same time maintaining animal welfare standards in their flocks. Hence, veterinarians active in that field need to offer improved services appropriate to the management system prevailing in respective sheep farms and take into account specific aspects of health management in each farm [1]. A good health management plan is the foundation for a thriving farm, and with that approach, veterinarians will support farmers, developing a mutually beneficial collaboration. Lacasta et al. [1] and Scott et al. [2] have indicated that a veterinary flock health plan is needed to serve several purposes: reduction of economic losses due to disease, improvement of farm capacity due to increased production, and reduction of adverse welfare effects of diseases.

Vaccinations constitute an integral part of health management programs in sheep flocks. Vaccines have contributed to the prevention, control and eradication of diseases more than any other tool available to veterinary medicine [3]. Classical vaccine development and production technologies have been used successfully for decades against a number of bacterial or viral diseases in animals. Various types of vaccines exist, which, among others, include ones consisting of whole cell cultures (attenuated or inactivated), toxoid vaccines, DNA vaccines or their combinations [3].

Bacterial mastitis is an important disease of sheep, which leads to increased financial losses and contributes to reduced welfare of affected animals [4]. In a recent countrywide study in Greece, the prevalence of subclinical mastitis was found to be 26% [5]. In Italy, relevant reports provided widely varying results: 10% to 50% frequency of bacterial isolation [6]. In Turkey, the prevalence of subclinical mastitis was found to be 18.5% [7]. In Spain, in an older study [8], the prevalence of subclinical mastitis was found to be 34%.

The disease has a multi-faceted aetiology. It is caused by various microbial agents, most frequently staphylococci (*Staphylococcus aureus* or coagulase-negative staphylococci) [6]. *S. aureus* is the most common clinical mastitis-associated pathogen in sheep [5,9]; Bergonier et al. [10] have indicated that *S. aureus* was the major pathogen responsible for the clinical disease, isolated from sporadic cases or outbreaks of the disease. The organism is considered to be responsible for about 40% of clinical cases in ewes suckling lambs and 80% of clinical cases in milking ewes [11,12,13]. The importance of coagulase-negative staphylococci as aetiological agents of subclinical mastitis has been repeatedly reported [14,15]; for example, in a recent, very extensive field investigation in dairy sheep, staphylococci have been found to account for approximately 70% of cases of subclinical mastitis [5]. *Mannheimia haemolytica* is of significance in ewes suckling lambs, hence of greater interest in meat production type flocks. Other pathogens (e.g., *Escherichia coli*, streptococci) are of limited importance and constitute, cumulatively, less than 15% of agents isolated from cases of mastitis [6].

Moreover, many factors also predispose ewes to the disease. These include genetic, ethological, management, nutritional, environmental and various other factors [16]. This makes it evident that mastitis is a multifactorial disease and, hence, its control also requires many approaches at various levels.

The present review will discuss the application of vaccination for the prevention of bacterial mastitis in ewes, performed as part of health management schemes. It is noteworthy that the first attempt to prevent mastitis by immunisation of sheep dates back to the early 20th century [17], underlining the significance of the disease, as well as the long-standing efforts to control it by means of immunisation of sheep.

## 2. General Principles of Vaccination against Mastitis in Sheep

As with all vaccinations, it is imperative to confirm that the animals to be vaccinated are healthy; thus, they would elicit a protective immune response. Vaccination is an active process where the immunological system of the animal is requested to mount an adequate response against the antigen administered. The basis of vaccination is the enhancement of acquired/specific immunity in the mammary gland. Vaccination aims at the recognition of specific determinants of a pathogen that activate a selective response leading to bacterial elimination [18].

Investigation of immunological responses in the mammary gland of ewes against causal organisms and clarification of the role of the various immunological components supports the attempts to immunise ewes against mastitis by enhancing immunity and potentiating responses to treatment with antibiotics. For example, it has been shown that local (intramammary) administration of immunogens (e.g., inactivated *Staphylococcus aureus*) in non-lactating ewes enhanced kinetics of neutrophil influx with no involvement of complement in the immunological response [18,19].

The primary protective role in mammary infections is played by cellular-type defences [19]. Mammary epithelial cells and leucocytes contribute significantly to the potential clearance of bacterial infections by performing phagocytosis, secreting antibacterial proteins and generating a variety of inflammatory mediators.

Additionally, immunoglobulins may leak into the mammary gland from blood (IgG1), may be produced locally by antigen-activated plasma cells (IgA, IgM) or may appear by either pathway (IgG2) [20,21,22]. Wellnitz et al. [23] have indicated that a possible intramammary increase of IgG1 and IgG2 is controlled and compound-specific, following various patterns and not exclusively an unspecific type of leakage. Low-level selective transport of plasma-derived IgE into mammary secretion of the ovine mammary gland, which may be augmented by low-level local production of IgE in the gland, has been also demonstrated [24]. Among them, IgG1, IgG2 and IgM can act in opsonizing bacteria [25].

In the mammary gland, the complement system participates in the immune defence, in evoking and controlling the inflammatory process, in participating in bacterial opsonisation and presentation, in recruiting leucocytes and in the direct killing of pathogens [26,27]. However, it is noteworthy that in the mammary gland, the classical pathway is not functional [28], whilst the alternative complement pathway is not as effective as in other tissues [28].

Naturally-occurring mammary infections can elicit a moderate immune response, which can lead to a reduction in the severity of subsequent infections and a lightly increased ability of the mammary gland to deal with them [29]. Staphylococci are part of the microbiota of the host skin; hence they co-evolute with their hosts, and in cases of mammary infection, the immune response may possibly lead toward tolerance [29], whilst further immune responses to commensals are calibrated to protect tissue homeostasis [30].

## 3. Application of Anti-Mastitis Vaccination in Sheep

### 3.1. Vaccines against Staphylococcal Mastitis

Initially, older technology, inactivated vaccines containing whole bacterial cells and/or staphylococcal toxoids, have been developed and used for the prevention of mastitis in ewes [31]. These products offered mostly a reduction in the severity of clinical signs rather than a reduction in the incidence risk of the disease.

The first attempt to develop a staphylococcal subunit vaccine for intramuscular administration to sheep was published by Amorena et al. [32]. The product included inactivated *S. aureus* and *S. simulans* whole cells, as well as *S. aureus* exopolysaccharide antigens presented within liposomes. The product has been found to lead in reducing the incidence risk of the disease after experimental staphylococcal infections; however, when the exopolysaccharide component had been omitted, no protection was evident against *S. aureus* strains [32].

In another study, the importance of adjuvants, specifically mineral oil or carbopol, within a vaccine containing inactivated *S. aureus* cells and α- and β-toxoids was assessed in sheep [33]. The vaccine was developed for administration around the supramammary lymph nodes. Two different adjuvants were evaluated: mineral oil and acrylic acid polymer resin (Carbopol). The use of mineral oil induced higher antibody titres against the toxoids, whilst the use of Carbopol induced higher titres against the bacterial exopolysaccharides.

Thereafter, Hadimli et al. [34] evaluated a staphylococcal whole-cell inactivated vaccine containing *S. aureus* and three coagulase-negative species for subcutaneous administration in the draining region of the supramammary lymph nodes of ewes. Nevertheless, the vaccine was found to offer limited protection against the disease.

Perez et al. [35] have described the induction of antibodies against poly-N-acetyl β-1,6 glucosamine exopolysaccharide, which is the main component of the extracellular biofilm matrix of staphylococci. Based on that principle, a vaccine has been produced, which made use of cell-free surface polysaccharides in various vehicles and contained free bacteria or bacteria embedded within their biofilm matrix with the use of various adjuvants. This immunological product was found to elicit an exopolysaccharide-specific antibody response offering protection against *S. aureus* mastitis [36]. Moreover, this vaccine contributed to the prevention of slime production by staphylococcal isolates and consequent biofilm-formation by these bacteria, thus limiting their expansion and dissemination within and outside the mammary gland and has now been licenced in the European Union (Vimco^®^; Hipra Animal Health, Girona, Spain) for administration to ewes. Some more recent publications have presented various aspects of its efficacy; for example, Vasileiou et al. [37] have documented efficacy in reducing the incidence risk of staphylococcal mastitis in sheep during a controlled field trial, offering protection against *S. aureus* and coagulase-negative staphylococci, whilst a later study has also confirmed efficacy against experimentally induced staphylococcal intramammary infection in ewes [38]. The presence of anti-staphylococcal biofilm antibodies in the blood of ewes after vaccination with this fully licenced vaccine has been shown to occur in the controlled field [37] or experimental [38] studies, as well as in spontaneously collected field samples [39]. Increased antibody titres were found to be associated with lower severity of mammary lesions and consequently with higher milk yield [38], as well as with lower prevalence of subclinical mastitis caused by biofilm-forming staphylococci [39].

The efficacy of a multivalent whole-cell staphylococcal vaccine for subcutaneous administration to ewes has been assessed by Alekish et al. [40]. The authors have reported that it led to a non-significant reduction in new cases of mastitis in vaccinated animals, with no effects on milk production or composition.

A different approach to the development of anti-staphylococcal vaccines in sheep has been presented by Longheu et al. [41]. These authors proposed new immunogens as candidates for vaccine development against *S. aureus*, specifically four cellular antigens (the proteins pyruvate kinase, elongation Factor Tu, dihydrolipoyl dehydrogenase and alpha-keto acid dehydrogenase) and three secreted antigens (bifunctional autolysin and the two components of the Panton-Valentine leucocidin, lukF-PV/lukM). The authors suggested that such a vaccine might be useful against mastitis caused by non-biofilm-forming isolates of *S. aureus*.

It is also noteworthy that often autogenous staphylococcal vaccines are produced. Such vaccines are for limited use in specific flocks and do not have wider applicability. There are no reports of their efficacy, as, obviously, they are produced for ‘tailor-made’ administration in flocks with the perceived problem of staphylococcal mastitis without first being evaluated for efficacy. Autogenous vaccines would be effective against one or a limited number of staphylococcal strains and might cause adverse reactions, especially if oily adjuvants are added. Azara et al. [18] have studied and presented the detailed characteristics of staphylococcal strains included in ovine anti-mastitis autologous vaccines produced in Italy, but not the clinical efficacy of these vaccines.

### 3.2. Vaccines against Mastitis Caused by Other Pathogens

Vaccines developed against *M. haemolytica* mastitis have included an autogenous vaccine developed by Kabay and Ellis [42], which, after single intraperitoneal administration to ewes, led to a reduction in the incidence risk of mastitis in a non-dairy sheep flock. More recently, it has been reported that the administration of a vaccine already licenced against respiratory infections of lambs conferred a protective effect against *M. haemolytica* mastitis in suckling ewes [43]. The protection was found to be complete but short-term (seven days) and offered only after direct intramammary administration of the product.

The vaccine discussed hereabove, as evaluated by Alekish et al. [40], also included *Streptococcus* spp. and *Trueperella pyogenes* antigens.

Finally, Leitner and Krifucks [44] have presented a murine model for the development and initial assessment of a vaccine specific for the prevention of *Pseudomonas*-associated mastitis. That vaccine was produced specially for use in sheep flocks with that particular problem.

## 4. Application of Anti-Mastitis Vaccination within the Frame of Flock Health Management

In mastitis, a disease caused by various bacterial pathogens, vaccinations should focus on preventing the most frequently occurring ones, i.e., staphylococci. Indeed, most currently licenced vaccines for the prevention of mastitis are anti-staphylococcal vaccines. Vaccines active against other pathogens would be of little benefit for general use in flocks, as these are infrequent mammary pathogens.

The expected benefits of mastitis vaccination in sheep flocks include the following.

**Figure 1:**
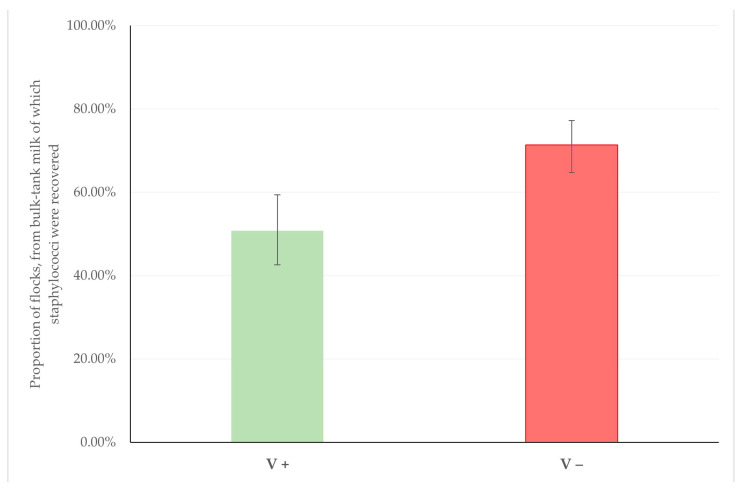
Proportion of flocks in which vaccination against staphylococcal mastitis was (V+) or was not (V−) applied, from the bulk-tank milk of which staphylococci were recovered (modified from Lianou et al. [45]) (bars indicate 95% confidence intervals).

**Figure 2:**
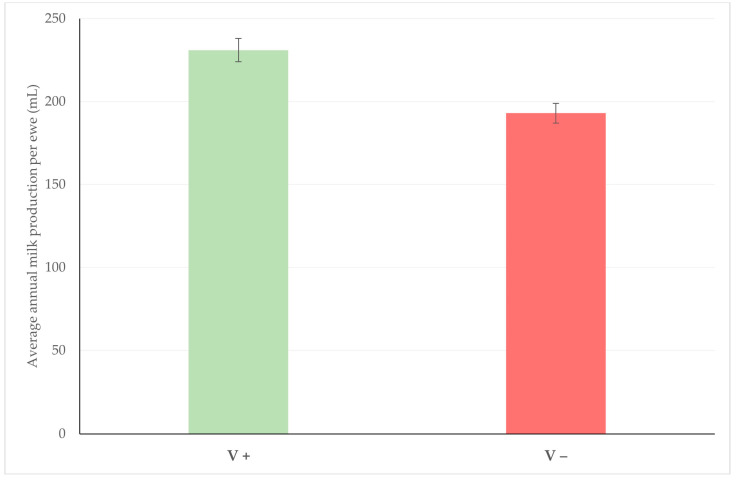
Average annual milk production per ewe among flocks in which vaccination against staphylococcal mastitis was (V+) or was not (V−) applied (modified from Lianou et al. [45]) (bars indicate standard error of the mean).

**Figure 3:**
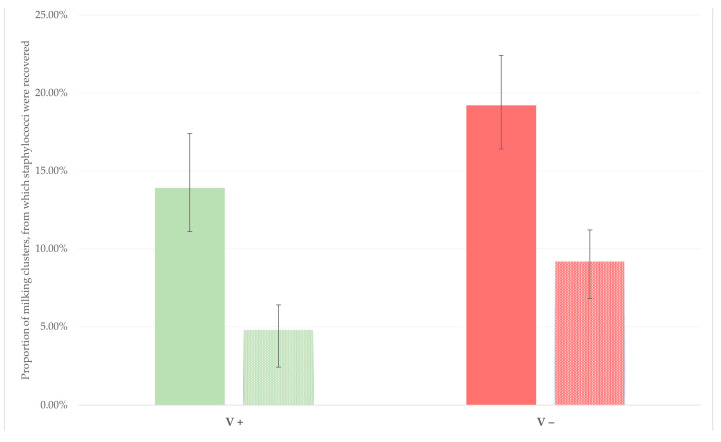
Proportion of swab samples from the upper (massif-filled bars) or the lower (pattern-filled bars) part of milking clusters from which staphylococci were isolated, in flocks in which vaccination against staphylococcal mastitis was (V+) or was not (V−) applied (modified from Michael et al. [46]) (bars indicate 95% confidence intervals).

1. Improved mastitis control, i.e., reduced incidence risk of clinical and subclinical mastitis. In this respect, the clinical studies (field and experimental work) carried out for licencing a vaccine, as well as any evidence becoming available will subsequently present data regarding the efficacy of a vaccine.
2. Improved milk quality: low somatic cell counts, optimum chemical composition, absence of staphylococci therein. In this respect, an association of the vaccination with a vaccine licenced against staphylococcal mastitis (Vimco^®^) with a reduced recovery of staphylococci from the farm bulk-tank raw milk was found in an extensive field study carried out in dairy sheep flocks (*n* = 325) in Greece. Among the 126 flocks in which sheep had been vaccinated against staphylococcal mastitis, staphylococci were recovered from 64 (50.8%), whilst among the 199 unvaccinated flocks, the bacteria were recovered from 142 (71.4%) (*p* = 0.0002) [45] (Figure 1).

3. Increased milk production, as the result of reduced incidence risk of the disease. In this respect, during the study referred to hereabove, it was found that milk production in flocks in which anti-staphylococcal mastitis vaccination was applied was higher than in which it was not performed: 231 ± 7 mL versus 193 ± 6 mL per ewe annually (*p* = 0.0001) [45] (Figure 2).

4. Reduced dissemination of mastitis-causing pathogens in the farm environment. In this respect, in an extensive field study carried out in dairy sheep flocks (*n* = 255) [46], an association was found between the vaccination with the same licenced vaccine against staphylococcal mastitis and the proportion of milking clusters from which staphylococci were isolated. From the 459 clusters sampled in sheep flocks where vaccination was performed, staphylococci were recovered from the upper part of 13.9% and the lower part of 4.8% of clusters; in contrast, higher recovery rates were seen among the 656 clusters sampled in sheep flocks where vaccination was not performed: 19.2% and 9.1%, respectively (*p* < 0.022) [46] (Figure 3).

5.. Reduction of antibiotic use in flocks, as cases of mastitis would be reduced and, consequently, the need for using antibiotics for their treatment would decrease. In this respect, animal vaccination is considered to be a valid and useful strategy for controlling diseases, recommended by guidelines for the prudent use of antibiotics in veterinary work [47] (Table 1).

In relation to the potential for reduction of antibiotic use in flocks, which is important within the One-Health context, many studies have shown such a reduction in cattle, pig, poultry and salmon farms [48,49].

It should be clarified that, as the result of the many predisposing factors to ovine mastitis, vaccination should be considered as one of many control measures that should be applied for the prevention of the disease. Other measures, e.g., post-milking teat disinfection, should also be assessed as part of udder health management in sheep flocks [5].

In general, vaccination against mastitis is performed during the final stage of pregnancy. The rationale for the administration of the vaccine at that time is to induce the protection of ewes starting at lambing, i.e., at the start of the lactation period when animals are at risk for developing the disease. Other practices carried out during the final stage of gestation, e.g., administration of anthelmintics, contribute to the improved immune response by ewes [50]. However, this approach poses a difficulty for the timely application of vaccinations during the final stage of gestation due to the short time available and the potentially large number of vaccines to administer (e.g., anti-clostridium vaccination, anti-orf vaccination, etc., as per requirements in individual flocks). For that, vaccinations should be prioritised by the attending veterinarian per flock, according to specific needs, and might be spread to a wider period, starting from the third month of gestation.

In conclusion, vaccination for mastitis is a useful, targeted preventive measure. Priority should be given to the administration of staphylococcal vaccines, as these organisms constitute the most frequent aetiological agents of clinical and subclinical mastitis. Whilst vaccination would contribute to reducing the incidence risk of mastitis; it should always be part of a general udder health management program.

## Figures and Tables

**Table 1:** Summary of expected benefits of mastitis vaccination in sheep flocks.

Expected Benefits
Improved mastitis control (reduced incidence of mastitis)
Improved milk quality (low somatic cell counts, absence of relevant bacteria)
Increased milk production
Reduced dissemination of mastitis pathogens (low excretion of staphylococci in milk)
Reduction of antibiotic use in flocks

## Data Availability

Not applicable.
